# Increased Infiltration of Extra-Cardiac Cells in Myxomatous Valve Disease

**DOI:** 10.3390/jcdd2030200

**Published:** 2015-07-24

**Authors:** Kimberly Sauls, Katelynn Toomer, Katherine Williams, Amanda J. Johnson, Roger R. Markwald, Zoltan Hajdu, Russell A. Norris

**Affiliations:** 1 Department of Regenerative Medicine and Cell Biology, Children’s Research Institute, Medical University of South Carolina, 171 Ashley Ave, Charleston, SC 29425, USA; E-Mails: saulsk@musc.edu (K.S.); toomerk@musc.edu (K.T.); willilin@musc.edu (K.W.); johnaj@musc.edu (A.J.J.); markwald@musc.edu (R.R.M.); 2 Department of Bioengineering, Clemson University, 200 C Patewood Drive, Greenville, SC 29615, USA; E-Mail: zhajdu@clemson.edu

**Keywords:** Filamin-A, myxomatous valve disease, hematopoietic cells, macrophage

## Abstract

Mutations in the actin-binding gene *Filamin-A* have been linked to non-syndromic myxomatous valvular dystrophy and associated mitral valve prolapse. Previous studies by our group traced the adult valve defects back to developmental errors in valve interstitial cell-mediated extracellular matrix remodeling during fetal valve gestation. Mice deficient in *Filamin-A* exhibit enlarged mitral leaflets at E17.5, and subsequent progression to a myxomatous phenotype is observed by two months. For this study, we sought to define mechanisms that contribute to myxomatous degeneration in the adult *Filamin-A*-deficient mouse. *In vivo* experiments demonstrate increased infiltration of hematopoietic-derived cells and macrophages in adolescent *Filamin-A* conditional knockout mice. Concurrent with this infiltration of hematopoietic cells, we show an increase in Erk activity, which localizes to regions of MMP2 expression. Additionally, increases in cell proliferation are observed at two months, when hematopoietic cell engraftment and signaling are pronounced. Similar changes are observed in human myxomatous mitral valve tissue, suggesting that infiltration of hematopoietic-derived cells and/or increased Erk signaling may contribute to myxomatous valvular dystrophy. Consequently, immune cell targeting and/or suppression of pErk activities may represent an effective therapeutic option for mitral valve prolapse patients.

## 1. Introduction

Mitral Valve Prolapse (MVP) is a common, degenerative cardiovascular disease that occurs in 2.4% of the population. MVP occurs when enlarged and weakened mitral leaflets billow back into the left atrium and fail to properly coapt during ventricular systole, which can lead to mitral regurgitation, congestive heart failure, and even sudden cardiac death. Histologically, the valves are characterized as myxomatous due to progressive fragmentation of collagen, expansion of the proteoglycan-rich spongiosa, and increased cell proliferation. These molecular and cellular changes result in enlarged mitral leaflets that are mechanically weakened over time and become ineffective at preventing the backflow of blood. There are no known nonsurgical cures for MVP, and, as such, it is currently the most common cause of mitral valve surgery. This is likely due to poor understanding of disease etiology and progression. However, previous studies have demonstrated that mutations in the *FLNA* (*Filamin-A*) gene are causal to a rare X-linked myxomatous valvular dystrophy in humans and may provide critical clues to disease inception and pathogenetic manifestation.

The Filamin family consists of three homologous proteins: A, B and C. Filamins A and B are widely expressed, while Filamin-C expression is predominantly restricted to cardiac and skeletal muscle. Filamin exists as a homo- or hetero-dimer where it functions in crosslinking actin filaments, mechano-sensing through cell surface-bound integrins, and by means of these interactions regulates cell adhesion and migration [1,2]. Filamin-A has been shown to directly interact with a variety of intracellular proteins and can function as a scaffold for second messengers in signal transduction. Specifically, Filamin-A has been shown to directly bind the TGFβ signaling mediators Smad and R-ras [3,4]. Complete genetic removal of Filamin-A in the mouse results in embryonic lethality by embryonic day 14 due to vascular hemorrhaging and severe cardiac malformations [5,6]. Our recent studies have shown that Filamin-A is highly expressed in the mitral valve during development and is significantly diminished after birth, suggesting an important role for Filamin-A during valve development [7]. We previously reported the developmental importance for Filamin-A in the valves by generating conditional knockout mice. In these studies, Filamin-A was genetically removed from endothelial-derived tissue of the atrioventricular valves, and resulted in enlarged mitral leaflets that progressed to myxomatous degeneration by two months of age. The developmental defects were attributed to alterations in valvular interstitial cell contractility and impaired ability to remodel the maturing valve matrix. These studies importantly defined a developmental etiology for myxomatous valves and have provided a clinically relevant model to study mechanisms of disease pathogenesis. Thus, in this report we build on these previous studies and interrogate postnatal mechanisms by which myxomatous degeneration occurs using the Filamin-A-deficient mouse as the model for the disease.

## 2. Experimental Section

### 2.1. Gene Targeted Mice

All mouse experiments were performed as previously described [8]. Briefly, female mice homozygous for a conditional “floxed” allele of *FLNA* (*Filamin-A^f^*^/*f*^) were bred with male transgenic mice expressing Cre under control of the *Tie2* promoter (*Tie2^Cre^*^(*+*)^), which will target valve endocardium and endocardial-derived mesenchyme, resulting in a (*Tie2^Cre^*^(*+*)^; *Filamin-A^f^*^/*y*^) conditional knockout (cKO) mouse [9].

### 2.2. Histology, Immunohistochemistry/Immunofluorescence

Standard histological and immunochemical methods were used as previously described [8]. All mouse immunofluorescence was performed on 5-micron sections of 2-month-old adolescent wildtype (WT) and cKO mice. Human immunofluorescence was performed on 5-micron sections of posterior leaflet from the mitral valve of normal (control) and diseased (myxomatous) tissue. Human tissues were obtained from surgical specimens as part of a Leducq transatlantic network. All samples were harvested by offsite partners and were fixed, embedded and sectioned in their various laboratories. Sections were sent to our group at the Medical University of South Carolina (MUSC) for histological assessment. Consent and Institutional Review Board (IRB) approval for these studies is in place at partnering institutions. For human tissue analyses, 3 control and 5 myxomatous valves were analyzed. Control valve biopsies were obtained from patients who perished as a result of either subarachnoid hemorrhage or intracranial bleeds. Myxomatous valves were surgical cases obtained from patients who fit diagnostic criteria of myxomatous degeneration and mitral valve prolapse. This was defined by >2 mm atrial leaflet displacement in a parasternal long-axis view as well as >5 mm valve thickness. All patient samples involved in the study were men greater than 50 years of age. For immunohistochemistry (IHC): Antigen retrieval was performed for 10 min using antigen unmasking solution (Vector Laboratories, Burlingame, CA, USA, Cat#H-3300) by pressure cooker (Cuisinart, Stamford, CT, USA). Antibodies and their dilutions used for immunological experiments include: Collagen I (MD Biosciences, St. Paul, MN, USA, Cat#203002, 1:200), HaBP (EMD Millipore, Billerica, MA, USA, Cat#385911, 1:200), pERK1/2 (Cell Signaling Technology, Danvers, MA, USA, Cat#4370, 1:50), MMP2 (Abcam, Cambridge, MA, USA, Cat#ab37150, 1:250), MF20 (DSHB, Iowa City, IA, USA, Concentrate, 1:50), Ki67 (Abcam, Cat#ab16667, 1:250), Filamin-A (Abcam, Cat#ab76289, 1:250), MMP3 (Abcam, Cat#ab38907, 1:250), Versican (gift from Stan Hoffman, Medical University of South Carolina, 1:250), MMP7 (Abcam, Cat#ab38996, 1:500), CD45 (Abcam, Cat#ab10558, 1:100, for mouse), and CD45 (EMD Millipore, Cat#05-1410, 1:100, for human). For fluorescent detection of the primary antibodies, Alexa fluor 568 and Alexa fluor 488 secondary antibodies were used (Life Technologies, Rockville, MD, USA). Hyaluronan-binding protein (HaBP) was visualized by streptavidin-fluorescein (Life Technologies, Cat#S-32354, 1:100). Nuclei were counterstained with Hoechst (Life Technologies, Cat#H3569, 1:10,000) for 10 min and slides were coverslipped with SlowFade mounting medium (Life Technologies, Cat#S36937). Images were acquired with a Leica fluorescent microscope (Leica Biosystems, Buffalo Grove, IL, USA); *n* > 3 for each experiment. 

### 2.3. Western Blotting

Isolated mitral leaflets from 2-month old mice were placed in SDS-PAGE buffer and boiled at 95 °C for 7 min before being run on a 4%–20% gradient polyacrylamide gel by electrophoresis (Biorad, , Cat#456-1094). A single leaflet per lane was used for Western blot experiments. Gels were transferred to a nitrocellulose membrane (Biorad, Cat#170-4158) and blocked for 1 h in 5% block (Biorad, Cat#170-6404) dissolved in 1X tris-buffered saline with Tween20 (BDH Chemicals, Poole Dorset, UK). Primary antibodies were incubated at (1:1000) dilution overnight at 4 °C. Primary antibodies used were pERK1/2 (Cell signaling, Cat#4370), pJNK1/2 (Cell Signaling, Cat#9255S), Erk (Cell Signaling, Cat#4695), JNK (Cell Signaling, Cat#9258S), MMP2 (Abcam, Cat#ab51125), MMP13 (Abcam, Cat#ab51072), and Actin (EMD Millipore, Cat#mab1501). Appropriate HRP-conjugated secondary antibodies were used at (1:10,000) in 5% block and incubated for 1 h at room temperature. Protein was detected using a chemiluminescent substrate (Life Technologies, Cat#34095). The entire process of tissue extraction to detection occurs within 18 h. We have determined that the rapidity of these experiments is critical in obtaining reproducible Western blots from single murine leaflets due to the small size and relative amounts of protein present in the extracts. Exact numbers of replicates are denoted in figure legends.

### 2.4. Statistics

Statistical significance was determined using a student’s *t*-test (two-tailed, type 2), with significance (*p* < 0.05). Statistical data are presented as standard deviations from the mean.

## 3. Results and Discussion

### 3.1. Adolescent Filamin-A cKO Mice Exhibit Myxomatous Changes by Two Months

**Figure 1:**
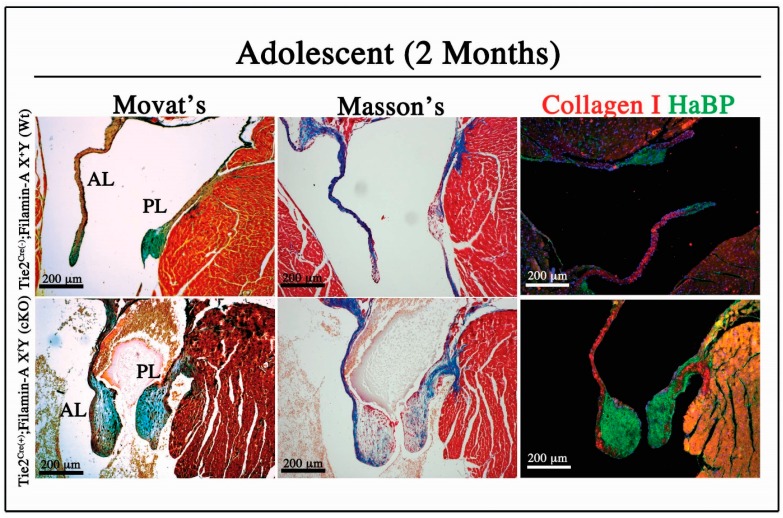
Filamin-A-deficient Mice Exhibit Myxomatous Mitral Leaflets. Adolescent 2-month-old mice were analyzed by Movat’s Pentachrome, Masson’s Trichrome, and Immunofluorescence for Collagen I (**red**) and Hyaluronan-binding protein (HaBP) (**green**). Movat’s: Collagen (**yellow**), proteoglycans (**blue**), elastin/nuclei (**black**), muscle (red). Masson’s: Collagen (blue), nuclei (black), muscle (red). Filamin-A-deficient mice exhibit increased proteoglycan content and disrupted matrix organization characteristic of myxomatous degeneration.

As a model for myxomatous valvular dystrophy, the *Filamin-A^f^*^/*f*^ mouse was bred onto the *Tie2^Cre^*^(+)^ background to genetically remove Filamin-A from all endothelial and endothelial-derived cells [8]. Since the mitral valve is largely derived from endothelium, the *Tie2^Cre^*^(*+*)^; *Filamin-A^f^*^/*y*^ cKO mouse, previously described by our group, provides a model to study mechanisms that lead to the progression of myxomatous degeneration [8]. Histological analysis demonstrates enlarged mitral leaflets in the cKO mouse compared to the thin and elongated leaflets of the WT by two months of age (Figure 1). Movat’s, Masson’s, and immunohistochemistry (IHC), for collagen I and HaBP, demonstrate characteristics of myxomatous leaflets in the cKO, with an increase in the proteoglycan-rich regions and disruption of normal matrix organization.

### 3.2. Filamin-A-Deficient Mice Exhibit Increased Erk Signaling Concurrent with Increased MMP Expression and Cell Proliferation

**Figure 2:**
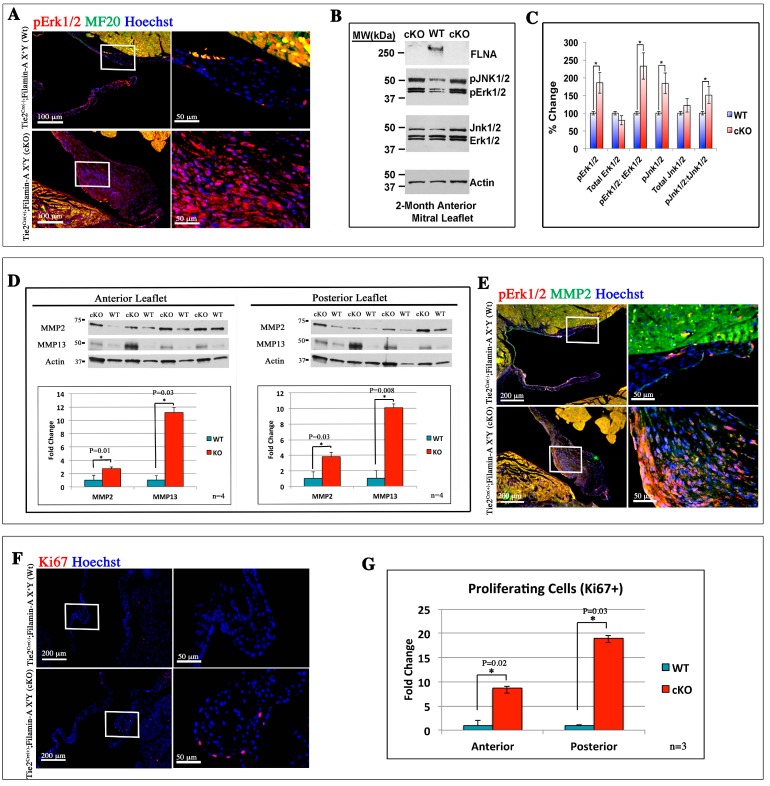
Erk1/2 Signaling, MMP expression, and Cell Proliferation are Increased in the Filamin-A cKO (**A**) IHC for phospho-Erk1/2 (**red**), MF20 (**green**), and nuclei (**blue**) demonstrate increased pErk accumulation in the 2-month-old cKO mitral valve; (**B**) Isolated anterior mitral leaflets from 2-month-old animals were analyzed individually by Western blot and demonstrate increased pErk1/2 and pJNK in relation to total protein, in the cKO compared to the WT; (**C**) Quantification of Western blot with * *p* < 0.05, *n* > 4; (**D**) Isolated anterior and posterior leaflets of cKO animals show increased expression of MMP2 and MMP13 compared to WT animals. All significant values are * *p* < 0.05, *n* = 4; (**E**) Immunofluorescence shows co-localization of pErk1/2 (**red**) with MMP2 expression (green); (**F**) Proliferating cells marked by Ki67 (**red**) and nuclei (**blue**) were quantified; (**G**) in both anterior and posterior leaflets. All significant values are * *p* < 0.05, *n* = 3.

To determine pathogenic mechanisms that contribute to the enlarged and myxomatous mitral valve in the cKO mouse, we performed an *in vivo* analysis examining alterations in signaling. Filamin-A functions in the regulation of various growth signaling pathways, including TGFβ, through direct interactions with downstream signaling mediators [1]. We hypothesized that loss of Filamin-A would result in the disruption of Erk1/2 signaling and that this may be pathogenic in myxomatous degeneration. By Western blot quantification of phospho/total Erk1/2 and phospho/total JNK in 2-month-old isolated anterior leaflets, and by IHC, we demonstrate that cKO mice have increased pErk1/2 and pJNK in the mitral valve (Figure 2A–C). Concurrent with these signaling activities, we observe increases in expression of the matrix metalloproteinases MMP2 and MMP13 in both mitral leaflets of the cKO compared to WT littermates (Figure 2D). Additionally, pErk localizes to regions of high MMP2 expression, suggesting excessive pErk1/2 may be an upstream signal promoting MMP expression in the diseased valve (Figure 2E), consistent with previous reports [10]. In addition to MMP expression, nuclear translocation of phospho-Erk1/2 has been reported to promote cell proliferation thorough activation of cell cycle regulatory proteins [11]. Since we see increased Erk1/2 signaling in our cKO mouse, we examined proliferation by immunostaining for ki67, and quantified proliferating cells. As shown in Figure 2F,G, proliferation was significantly increased in the cKO compared to the WT in both anterior and posterior leaflets.

### 3.3. Increased Infiltration of Hematopoietic-Derived Cells in Myxomatous Valves Contribute to Disease

**Figure 3:**
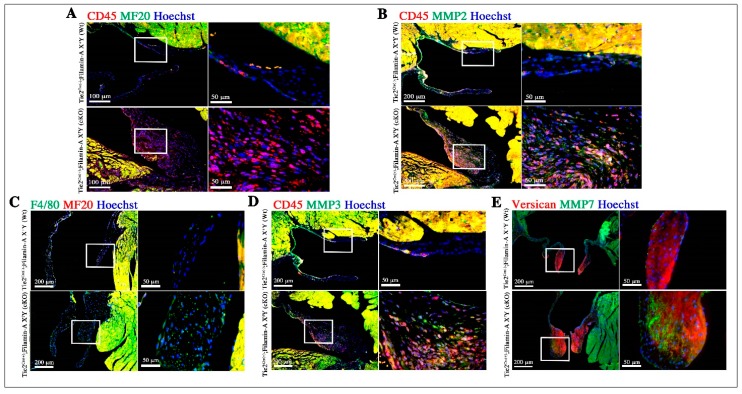
CD45-positve Cells Increased in the Filamin-A cKO mouse and may Contribute to Myxomatous Degeneration. (**A**) CD45-positive cells (**red**) are increased in the cKO compared to WT littermates; (**B**) CD45 cells (red) localize to MMP2 (**green**) expression in the posterior leaflet; (**C**) The macrophage marker F4/80 (green) is increased in the cKO posterior leaflet compared to the WT; (**D**) CD45-positive cells (red) express MMP3 and are localized to regions of secreted MMP3; (**E**) MMP7 (green) is increased in the CKO and co-localizes with versican expression (**red**).

To account for molecular changes (pErk, pJNK) and downstream consequences (MMP expression, proliferation) that contribute to disease in two-month-old cKO mice, the possibility that extra-cardiac cells may be promoting these pathogenic changes was examined. Hematopoietic cells have been shown to engraft into the murine adult mitral valve, and exhibit synthetic properties characteristic of fibroblasts as a part of normal valve homeostasis [12,13]. We hypothesized that in the Filamin-A model of myxomatous mitral valve disease, alterations in hematopoietic cell engraftment would be present in diseased valves and that these cells contribute to disease pathogenesis. Using CD45 as a marker for hematopoietic-derived cells, we observed an increase in the number of CD45-positive cells in the mitral leaflets of the cKOs compared to WT littermates (Figure 3A). CD45-positive cells engrafted primarily in the sub-endocardial region of the WT mouse, which is consistent with other reports in WT mice [12,13]. The cKO mice exhibited increased engraftment of hematopoietic-derived cells not only sub-endocardially, but also in the valvular interstitum [12]. To determine if these cells could be contributing to the myxomatous phenotype, we examined MMP expression as it related to the CD45-positive cells. Extracellular MMP2 localized to regions surrounding CD45-positive cells, suggesting these cells may be a significant source of MMPs (Figure 3B).

Hematopoietic-derived cells that express CD45 are highly plastic and have been shown to develop into fully differentiated macrophages [14,15]. Since we see an increase in CD45-positive cells, we used the pan-macrophage marker F4/80 to determine if macrophage infiltration is also increased. Histologically, we observe an increase in expression of F4/80 in the cKO compared to the WT. This correlation between increased CD45 and F4/80 expressing cells suggests infiltrating CD45-positive cells may also be macrophages (Figure 3C).

To determine if there is an association between extra-cardiac cells and pathogenic mechanisms of disease, further characterization by IHC was performed. Macrophages are known to express MMPs at high levels, and have been specifically associated with MMP3 and MMP7 [16]. Our CKO mice show increased expression of both MMP3 (Figure 3D) and MMP7 (Figure 3E). Additionally, elevated levels of MMP3 were found in regions enriched with CD45-positive cells in cKO valves (Figure 3D). MMP7 distribution was primarily localized to the proteoglycan-enriched region of the leaflets, indicating a potential role in proteoglycan cleavage (Figure 3E). This data further suggests CD45-positive cells present in the mitral valve are involved in matrix remodeling and degradation.

**Figure 4:**
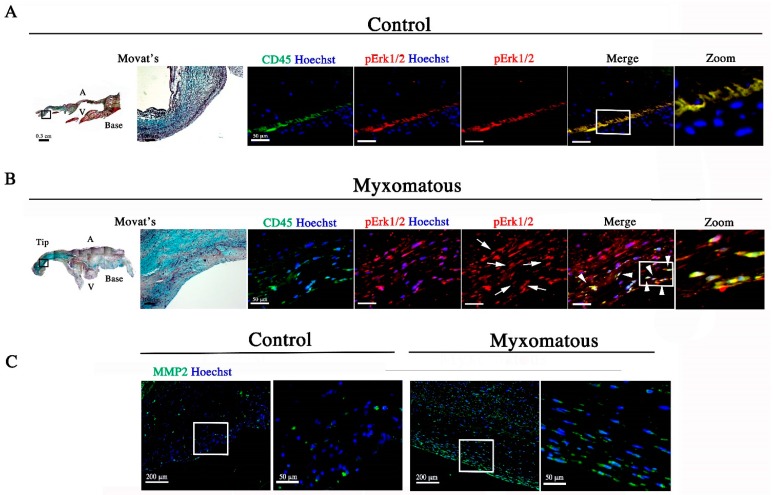
Myxomatous Human Mitral Leaflets Show Increased Infiltration of CD45-positive Cells. (**A**) IHC for CD45 (**green**) and pErk1/2 (**red**) and Movat’s depicting elastin/nuclei (**black**), collagen (**yellow**), proteoglycans (**blue**), in the posterior leaflet from a normal human mitral valve; (**B**) Movat’s and IHC in a myxomatous mitral leaflet form a human patient shows increased pErk1/2 (**red**) and CD45 cell (**green**) infiltration compared to control. CD45-positive cells also express pErk1/2 (**arrowheads**) and pErk1/2 is nuclear (**arrows**); (**C**) IHC for MMP2 (**green**) shows increased expression in the human myxomatous posterior leaflet. All images depict similar anatomical regions of the posterior leaflet.

To determine if the cellular and molecular changes observed in the cKO mouse are relevant to human disease, pErk, CD45, and MMP expression were examined in myxomatous and normal mitral valve tissue obtained from patients who underwent mitral valve surgery or autopsy respectively. In normal mitral tissue (control), pErk1/2 was present but restricted to the ventricularis layer and localized to the sub-endocardial region (Figure 4A). Myxomatous leaflets displayed increased pErk1/2 activity and in contrast to the control, pErk1/2 was active throughout all layers of the leaflet and was nuclear in localization compared to the primarily cytoplasmic expression in the control (Figure 4A,B). Additionally, CD45 positive cell infiltration was present in both the control and myxomatous leaflets, and these cells also express pErk1/2. Control leaflets exhibited restricted pErk1/2 activation in the region where CD45-positive cells reside. In contrast, myxomatous leaflets exhibit pErk1/2 activation not only in CD45-positive cells themselves, but also in proximal valve interstitial cells, suggesting the extra-cardiac cells may exhibit potential paracrine effects. MMP2 expression was also increased in the myxomatous valve compared to the control, and its localization was consistent with the pErk1/2 seen in the myxomatous valve (Figure 4C).

### 3.4. Discussion

Our results demonstrate that myxomatous changes occur by two months of age in the *Tie2^Cre^*^(*+*)^; *Filamin-A-X^f^Y* conditional knockout model of myxomatous mitral valve disease. Previous studies have established a link between increased growth factor signaling and syndromic forms of myxomatous mitral valve disease. For instance, patients with Marfan syndrome, a connective tissue disease that can result in myxomatous mitral valves and MVP, have indicated increased Erk1/2 signaling as a potential pathogenic mediator of the disease [17,18,19,20,21]. Marfan syndrome is caused by mutations in *FBN1* (*Fibrillin-1*), which result in excessive TGFβsignaling due to diminished binding of the latent form of the growth factor. This aberrant regulation of TGFβ results in alterations in pErk and pSmad2/3 activity accompanied by increased MMPs and cell proliferation [17]. As Filamin-A is known to interact with TGFβ signaling components, and mutations in FLNA cause a myxomatous valve disease that is similar to the mitral valve phenotype in Marfan syndrome, we hypothesized that Erk and Jnk signaling may also be elevated in the mitral leaflets of the Filamin-A deficient mouse. Indeed, our results presented here demonstrate increased Erk and Jnk activity in the mitral valve when Filamin-A is lost and that this signaling correlates with regions of MMP expression. Although TGFβ can initiate Erk signaling, we cannot directly tie increased Erk activity seen in our cKO mouse to TGFβ since many growth factors are known to be upstream of Erk signaling. These upstream signals include epidermal growth factor (EGF), fibroblast growth factor (FGF), and vascular endothelial growth factor (VEGF) [22]. It is yet to be determined whether these additional factors can activate the Erk/Jnk pathway in myxomatous valves. Regardless, upon activation Erk is translocated to the nucleus where it is known to promote expression of multiple genes, including MMPs [22]. The gelatinase MMP2, matrilysn MMP7 and the collagenase MMP13, were both significantly increased in the Filamin-A cKO valves. Increased expression of these enzymes are therefore likely candidates for contributing to myxomatous mitral valve degeneration and may contribute to the progressive deterioration of the structure and function of the valve over time. Additionally, previous studies have demonstrated that MMP-9 is also activated in cardiac and heart valve remodeling [23,24,25]. Together, these findings support a putative global stimulation of MMP expression and builds additional support for this class of enzymes in aberrant matrix destruction and remodeling in valve disease.

During normal valve homeostasis, hematopoietic-derived cells that exhibit synthetic processes characteristic of fibroblasts have been shown to engraft into the mitral valve [12]. In human myxomatous tissue, increased CD45-positive cells and blood-derived fibrocytes with matrix-altering abilities have also been observed [12,26,27]. Our data demonstrate an increase in hematopoietic cell engraftment in the context of human and murine myxomatous valve disease, and we show these cells may contribute |to myxomatous degeneration through matrix disruption (*i.e.*, MMP expression). In the mouse, regional localization patterns of hematopoietic cells differed between genotypes, where cells in the WT are localized sub-endocardially, while cells in the cKO integrate into the interstitial region of the leaflets. Correlated with infiltrating CD45-positive cells were significant increases in MMP2 expression in myxomatous leaflets, suggesting these cells may be a source. Additionally, increased MMP expression was observed throughout the leaflets and correlated regionally with CD45-positive cell infiltration, suggesting these cells may be a source of growth factors and promote myxomatous degeneration through paracrine mechanisms that alter the behavior of valvular interstitial cells.

To further characterize the hematopoietic cell population in the myxomatous mitral valve, we examined the possibility that these cells are monocytes/macrophages. In support of the idea that an immune response is initiated in myxomatous mitral valve disease, previous studies have identified serum increases in monocyte chemoattractant protein-1 (MCP-1) and additional increases in mast cells in dogs with myxomatous mitral leaflets [28,29]. In our cKO model, we observe an increase in macrophages in diseased mitral leaflets. This increase correlates with an increase in CD45-positive cells, suggesting these cells may also be macrophages. Expression of MMP3 by hematopoietic-derived cells further suggests these cells are TH-1 polarized M1 macrophages, which are classically associated with a pro-inflammatory phenotype and MMP expression [16]. To further support this, we show increased expression of MMP7, another macrophage-expressed metalloproteinase in M1 polarized macrophages. Expression of MMP7 preferentially corresponds to proteoglycan-rich regions of the mitral leaflets and is largely absent in the WT. Since MMP7 can cleave proteoglycans, this may be a mechanism by which proteoglycans are disrupted during disease. Both MMP-3 and MMP-7 degrade a number of ECM components including adhesive glycoproteins such as, laminin, fibronectin, tenascin, and some types of collagen [30]. Additionally, macrophages secrete factors that can have paracrine effects on the surrounding tissue. Previous studies have demonstrated this effect in dermal fibroblasts subjected to factors produced by M1 macrophages, where these cells show enhanced degradation properties in response to conditioned media [31]. Macrophages up-regulate MMPs in response to contact with different ECM components including collagen, laminin and fibronectin [32]. The mitral valve, which is composed of different matrix components, is an environment that would likely trigger up-regulation of MMPs in macrophages.

To determine if the molecular and cellular changes observed in the Filamin-A model of myxomatous valvular dystrophy are relevant to non-syndromic myxomatous disease in humans, we examined human resected mitral leaflets by IHC. Consistent with our murine studies, increases in pErk signaling were observed in human myxomatous posterior leaflets compared to normal tissue. Localization of pErk1/2 was confined to the ventricularis, sub-endocardial region of control leaflets and was primarily cytoplasmic, in contrast to the myxomatous leaflets, which displayed nuclear pErk1/2 activation throughout the entirety of the leaflet. Cellular populations of hematopoietic cells were increased in the human myxomatous leaflets and displayed co-expression of nuclear pErk1/2. MMP2 expression was also increased in myxomatous leaflets and observed in regions that encompass CD45 positive cells. Our results are consistent with previous reports that show increases in MMP2 activity in human mitral leaflets with myxomatous, floppy valves [33]. Taken together, our mouse and human data support the hypothesis that increased hematopoietic cell infiltration contributes to disease.

## 4. Conclusions

Loss of Filamin-A in the mitral leaflets of the mouse results in developmental defects in the structural architecture of the mitral valve. Post-natally, myxomatous degeneration occurs by two months of age and is accompanied by an increase in hematopoietic-derived cells, a sub-population of which are likely macrophages. These infiltrating extra-cardiac cells correlate with increased activation of Erk1/2 as well as putative downstream targets such as MMPs and genes that regulate and promote cell proliferation. Similar changes are observed in human myxomatous mitral valve tissue, whereby CD45-positive cells express nuclear pErk1/2. These murine and human studies suggest that infiltration of hematopoietic-derived cells and/or increased Erk1/2 signaling may be pathogenic to myxomatous valvular dystrophy. Consequently, immune cell targeting and/or suppression of pErk1/2 activities may represent an effective therapeutic option for mitral valve prolapse patients.
